# C-reactive protein-guided use of procalcitonin in COVID-19

**DOI:** 10.1093/jacamr/dlab180

**Published:** 2021-11-28

**Authors:** Rebecca Houghton, Nathan Moore, Rebecca Williams, Fatima El-Bakri, Jonathan Peters, Matilde Mori, Gabrielle Vernet, Jessica Lynch, Henry Lewis, Maryanna Tavener, Tom Durham, Jack Bowyer, Kordo Saeed, Gabriele Pollara

**Affiliations:** 1 Hampshire Hospitals NHS Foundation Trust, Hampshire, UK; 2 University Hospital Southampton NHS Foundation Trust, Southampton, UK; 3 School of Medicine, University of Southampton, Southampton, UK; 4 Royal Free London NHS Foundation Trust, London, UK; 5 University College London, London, UK

## Abstract

**Background:**

A low procalcitonin (PCT) concentration facilitates exclusion of bacterial co-infections in COVID-19, but high costs associated with PCT measurements preclude universal adoption. Changes in inflammatory markers, including C-reactive protein (CRP), can be concordant, and predicting low PCT concentrations may avoid costs of redundant tests and support more cost-effective deployment of this diagnostic biomarker.

**Objectives:**

To explore whether, in COVID-19, low PCT values could be predicted by the presence of low CRP concentrations.

**Methods:**

Unselected cohort of 224 COVID-19 patients admitted to hospital that underwent daily PCT and CRP measurements as standard care. Both 0.25 ng/mL and 0.5 ng/mL were used as cut-offs for positive PCT test results. Geometric mean was used to define high and low CRP values at each timepoint assessed.

**Results:**

Admission PCT was <0.25 ng/mL in 160/224 (71.4%), 0.25–0.5 ng/mL in 27 (12.0%) and >0.5 ng/mL in 37 (16.5%). Elevated PCT was associated with increased risk of death (*P = *0.0004) and was more commonly associated with microbiological evidence of bacterial co-infection (*P < *0.0001). For high CRP values, significant heterogeneity in PCT measurements was observed, with maximal positive predictive value of 50% even for a PCT cut-off of 0.25 ng/mL. In contrast, low CRP was strongly predictive of low PCT concentrations, particularly <0.5 ng/mL, with a negative predictive value of 97.6% at time of hospital admission and 100% 48 hours into hospital stay.

**Conclusions:**

CRP-guided PCT testing algorithms can reduce unnecessary PCT measurement and costs, supporting antimicrobial stewardship strategies in COVID-19.

## Introduction

The hyperinflammatory state in severe COVID-19 disease resembles but is rarely complicated by bacterial co-infections,^1^^,^^2^ hindering antimicrobial stewardship efforts that seek to minimize unnecessary antibiotic prescribing. Cross-sectional radiological changes lack specificity, microbiological investigations lack sensitivity, and culture-independent biomarkers such as C-reactive protein (CRP) and white cell count (WCC) only partially exclude co-infections,^1^^,^^3^ and procalcitonin (PCT) may provide additional diagnostic discrimination.^4^ In non-COVID-19 settings, elevated PCT is associated with bacterial than viral infections,^5^ and low PCT values (<0.5 ng/mL) can support cessation of antibiotics.^6^

Elevated PCT concentrations are observed in COVID-19 and are associated with poor prognosis,^7^^,^^8^ hindering the definition of cut-offs that diagnose bacterial co-infections in this context.^9^ Nevertheless, PCT values <0.5 ng/mL offer >95% negative predictive value (NPV) for microbiological evidence of bacterial co-infection in COVID-19,^10^ and PCT <0.25 or <0.5 ng/mL have been used to reduce antibiotic consumption without worsening clinical outcomes.^7^^,^^11–13^ PCT measurements are substantially more expensive than for CRP or WCC,^12^ and elevations in PCT, CRP and WCC can be concordant.^7^ Therefore, an important research objective is to predict scenarios where PCT values are invariably low, supporting the exclusion of bacterial co-infection and avoiding the expense of redundant PCT measurements. On the basis that absence of bacterial infections would induce minimal elevations in inflammatory markers, we sought to test the hypothesis that in COVID-19, low PCT concentrations could be predicted from low CRP or WCC values, thus informing cost-effective PCT testing algorithms in the routine clinical care of COVID-19.

## Patients and methods

### Patient selection

Patients were identified from electronic records and laboratory systems at Hampshire Hospitals NHS Trust. Inclusion criteria were >18 years old with a clinical syndrome compatible with COVID-19, SARS-CoV-2 confirmed detection by molecular diagnostic testing on nasopharyngeal swabs performed in the emergency department and requiring hospital admission between 5 March and 26 April 2020. The measurement of PCT, CRP and WCC during hospital stay was performed as standard clinical care. Significant microbiological identification was defined as the isolation of bacterial or fungal species from blood culture (excluding coagulase-negative staphylococci) or from sputum samples (excluding mixed respiratory flora or *Candida* spp), or detection of *Streptococcus pneumoniae* or *Legionella pneumophila* antigens in urine analyses.

### Data extraction and ethics

Patient demographics, comorbidities, microbiology results, admission to ICU and mortality data were collected retrospectively from hospital electronic health records. The study was approved by the Research and Development Department at Hampshire Hospitals NHS Trust, which stated that as this was a retrospective review of routine clinical data, formal ethics approval was not required.

### Statistical analysis

Baseline demographics between the cohorts stratified by PCT levels were compared by Chi-square test, except for age which was assessed by Kruskal-Wallis test. CRP and WCC levels between the cohorts were compared by Mann–Whitney tests. Geometric mean was used to define high and low CRP values. For pre-determined PCT and CRP cut-offs sensitivity, specificity, positive predictive value (PPV) and NPV were calculated. Analyses were performed using Microsoft Excel and GraphPad Prism.

## Results

We identified 299 adult patients diagnosed with COVID-19 admitted to Hampshire Hospitals NHS Trust between 5 March and 26 April 2020. Although measurement of PCT, CRP and WCC at baseline and daily during hospital admission was the standard of care, we focused on the 224 patients (75.0%) who received PCT testing on admission. Most patients (160/224, 71.4%) had admission PCT <0.25 ng/mL, whereas in 27 (12.0%) it was between 0.25–0.5 ng/mL and in 37 (16.5%) it was ≥0.5 ng/mL (Table 1). Admission PCT was not associated with differences in age, gender, ethnicity or admission to ICU, but elevated PCT was associated with increased risk of death during hospital admission (*P = *0.0004 by Chi-square test). Microbiological evidence of bacterial co-infection was absent in most patients (216/224, 96.4%), but was more common with elevated PCT values (*P < *0.0001 by Chi-square test). Blood cultures were collected in 92/224 (41%), sputum samples in 37/224 (16.5%) and urine antigens in 110/224 (49%) patients. Restricting our analyses to patients who underwent microbiological sampling preserved the association between positive microbiology and PCT levels (Table 1).

**Table 1.** Baseline demographics and clinical characteristics for patients included in the study, stratified by admission PCT concentrations

	PCT value (ng/mL) at admission			
Characteristics	<0.25 (*n = *160)	≥0.25–<0.5 (*n = *27)	≥0.5 (*n = *37)	*P* value
Age, median (range)	67 (26–97)	78 (18–92)	70 (30–97)	*P = *0.227
Gender, *n* (%)				
Male	89 (55.6)	21 (77.8)	22 (59.5)	*P = *0.096
Female	71 (44.4)	6 (22.2)	15 (40.5)	
Ethnicity, *n* (%)				
White	137 (85.6)	22 (81.5)	30 (81.1)	*P = *0.819
Asian	16 (10.0)	4 (14.8)	4 (10.8)	
Black	6 (3.8)	0 (0.0)	1 (2.7)	
Mixed	1 (0.63)	0 (0.0)	1 (2.7)	
Other	2 (1.25)	1 (3.7)	1 (2.7)	
ICU admission, *n* (%)				
Yes	26 (16.3)	6 (22.2)	12 (44.4)	*P = *0.078
No	134 (83.8)	21 (77.8)	25 (67.6)	
Microbiology, *n* (%) [%]^a^				
Blood culture	0/65 (0.0) [0.0]	1/9 (3.7) [11.1] *E. coli*	2/18 (5.4) [11.1] α-haem streptococci *Corynebacterium* sp.	*P < *0.0001^b^
Sputum culture	0/22 (0.0) [0.0]	1/5 (3.7) [20.0] *K. pneumoniae*	1/10 (2.7) [10.0] *M. morganii*	
Urine antigen	0/79 (0.0) [0.0]	0/9 (0.0) [0.0]	2/22 (5.4) [9.1] *S. pneumoniae* (×2)	
Death, *n* (%)				
Yes	35 (21.9)	14 (51.9)	17 (45.9)	*P = *0.0004
No	125 (78.1)	13 (48.1)	20 (54.1)	

a Percentage values are calculated both relative to all patients in the group (values in round brackets) and also relative only to those that underwent microbiological sampling (values in square brackets). *P* values represent statistical assessments of variation between each variable and the defined patient cohorts.

b For microbiology tests, the *P* value was unchanged when analyses were restricted to only patients that had undergone microbiological sampling. Age was compared by Kruskal-Wallis test and all other variables were compared by Chi-square test.

**Figure 1.**
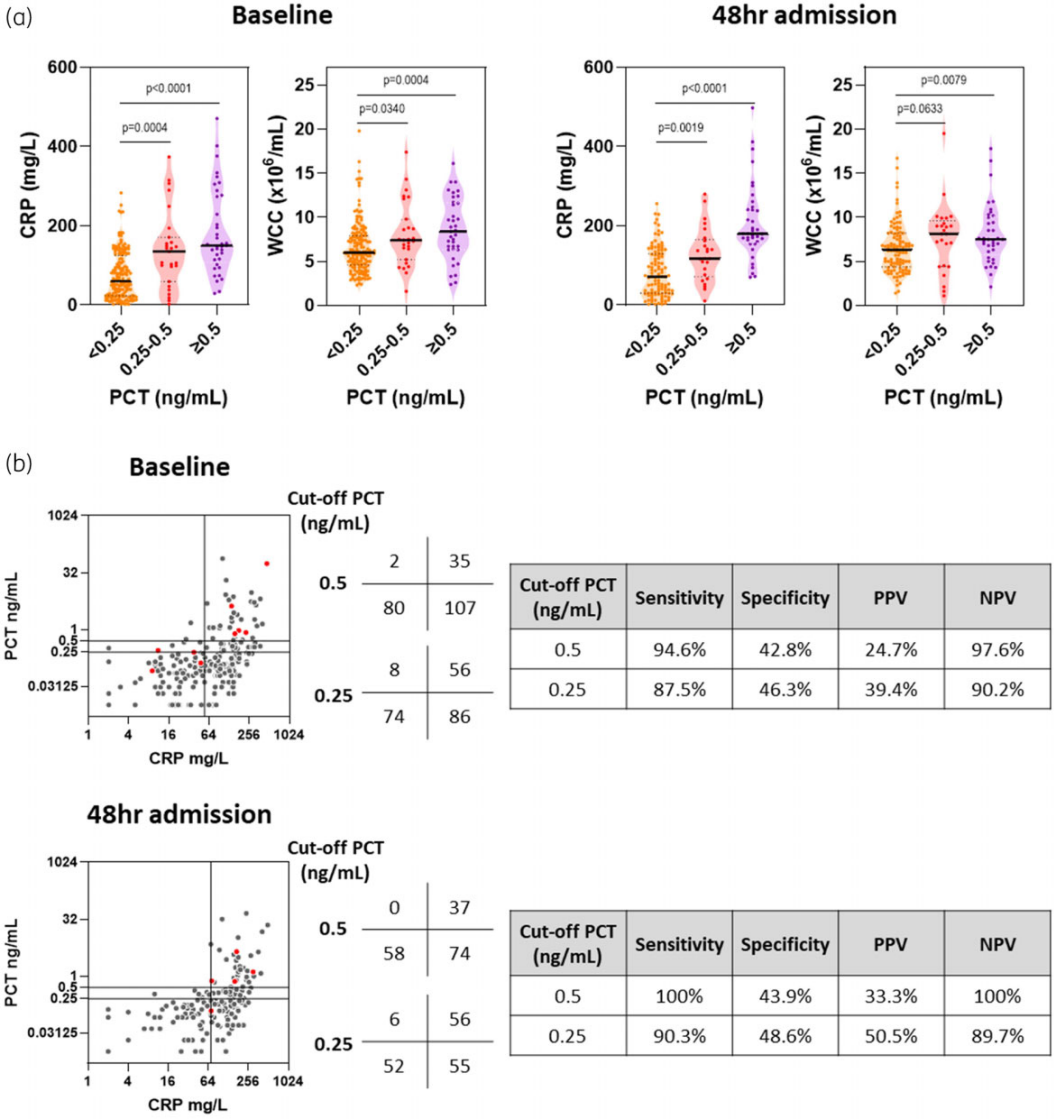
Association between inflammatory markers and the concentration of PCT in COVID-19. (a) Concentration of CRP or enumeration of WCC stratified by PCT concentrations. (b) Relationship between PCT and CRP concentrations. Scatter plot horizontal lines represent PCT concentration cut-off (≥0.25 or ≥0.5 ng/mL) and vertical lines represent geometric mean CRP for all patients at each timepoint (54 and 70 mg/L respectively). Total numbers of patients in each quadrant of scatter plots are shown in the adjacent table. Red dots indicate patients with significant microbiological findings. Sensitivity, specificity, positive predictive value (PPV) and negative predictive value (NPV) given were derived for elevated PCT determined by CRP concentrations derived at each timepoint for each PCT cut-off. Assessments were made at the time of hospital admission (‘baseline’) or 48 hours into hospital admission (‘48hr admission’). All *P* values derived using 2-tailed Mann–Whitney tests.

Next, we tested the hypothesis that PCT concentrations were related to CRP or WCC levels. Increases in PCT were closely associated with elevations in CRP and WCC, with CRP differences observed even as PCT values rose from <0.25 to 0.5 ng/mL (Figure 1a). To determine whether this relationship between inflammatory markers was generalizable, we tested the same hypothesis in the 169 patients who remained resident 48 hours after admission. Samples collected at this time revealed similar findings, with elevations in PCT again closely associated with rising WCC values and especially CRP concentrations (Figure 1a).

Given PCT was most closely related to CRP at both timepoints, we performed pairwise comparisons of these variables, testing the hypothesis that low CRP levels could predict low PCT concentrations. We used the geometric mean CRP for the entire patient population at each timepoint as cut-offs of low and high CRP (54 and 70 mg/L respectively) (Figure 1b). Although elevated PCT levels were almost exclusively associated with high CRP levels, with greatest sensitivity seen with a PCT cut-off of 0.5 ng/mL (94.6% and 100% at baseline and 48 hours into admission, respectively); not all patients with high CRP levels demonstrated elevated PCT values (all PPV were ≤50%) (Figure 1b). Positive microbiological findings were rare, but for those with high CRP, almost all had high PCT values irrespective of the cut-off used (Figure 1b). Most strikingly, low CRP levels observed in 82/224 (36.6%) and 58/169 (34.3%) of patients at baseline and 48 hours into hospital admission, respectively, were strongly predictive of low PCT values at both timepoints, with the greatest NPV seen with a PCT cut-off of 0.5 ng/mL (97.5% and 100%, respectively) (Figure 1b). Our use of the geometric mean cut-offs sought to better reflect the cohort’s CRP distribution and avoid skewing by outliers, but using median CRP as alternative cut-offs at each timepoint (79 and 91 mg/L) marginally reduced the NPV to 94.3% and 96.0% using a PCT cut-off of 0.5 ng/mL (Figure S1, available as Supplementary data at *JAC-AMR* Online).

## Discussion

Bacterial co-infections in COVID-19 are infrequent, creating a need to minimize excessive antibiotic prescribing and selection for resistance. WCC and CRP have limited discriminatory capacity,^1^ and PCT has been increasingly used to provide more diagnostic certainty, with several studies using low PCT values, which support the exclusion of bacterial co-infections, to safely reduce antibiotic prescribing.^7^^,^^11–13^ Our findings reveal that low PCT values, especially <0.5 ng/mL, can be predicted from low levels of routinely measured CRP, avoiding the associated costs of PCT testing in over one-third of patients. We propose that CRP-led algorithms may provide a cost-effective means to deploy PCT in the care of COVID-19 patients, removing the costs associated with redundant testing for this biomarker.

The natural history of PCT in COVID-19 remains unknown,^9^ and previously defined cut-offs to discriminate viral and bacterial infections may not be applicable,^5^^,^^6^ limiting the use of PCT to confirm bacterial co-infections and initiate appropriate antibiotic treatment. Nevertheless, we confirm previous observations that elevated PCT is associated with worse clinical outcomes.^7^^,^^8^ Given the rarity of bacterial co-infections, elevated pathological cytokine activity alone may be a key driver of PCT secretion in severe COVID-19.^14^^,^^15^ However, high CRP levels were associated with marked heterogeneity in PCT, and the highest PCT levels may also reflect genuine bacterial co-infection, which might be underestimated by the diagnostic limits of microbiological testing. Larger prospective studies and randomized controlled trials will define the relationship between PCT and bacterial co-infections in COVID-19 to guide antibiotic prescribing.^4^

Strengths of our study included routine PCT measurements in the care of this unselected COVID-19 cohort during the first pandemic wave in the UK, reproducibility of the findings at both timepoints and use of data-driven CRP thresholds that yielded comparable findings using either geometric mean or median values. We acknowledge the retrospective, single-centre nature of the study, which limits generalizability of the exact CRP cut-offs to different patient populations and testing laboratories, although our statistical approaches remain applicable for use at other centres. Microbiologically confirmed bacterial co-infections were rare but we acknowledge these investigations lack diagnostic sensitivity,^16^ and were not, or could not, be performed in all patients (e.g. in the absence of sputum production). Combined with the modest sample size and absence of information on pre-hospital antibiotic prescriptions, we were not able to define the true relationship between PCT and bacterial co-infections in COVID-19, highlighting the importance of not using PCT measurements alone to guide antibiotic prescribing.^10^^,^^17^ However, similar to others,^18^ we observed the relationship between inflammatory markers to be consistent throughout the hospital stay, indicating that inferred or measured PCT may be most useful in discontinuing antibiotics rather than withholding their initiation on admission, a timepoint accompanied by greater diagnostic uncertainty. Finally, liver dysfunction and immunomodulators (e.g. dexamethasone or tocilizumab) may limit CRP elevations in some bacterial co-infections.^19^^,^^20^ In these scenarios, it may not be appropriate to extrapolate low PCT values from CRP readings.

In conclusion, CRP levels can predict settings in which measurements of PCT will be low and therefore redundant. In this way, CRP-guided PCT testing algorithms can both reduce costs and support antimicrobial stewardship strategies in COVID-19.

## Funding

This study was carried out as part of our routine work.

## Transparency declarations

None to declare.

## Supplementary data

Figure S1 is available as Supplementary data at *JAC-AMR* Online.

## Supplementary Material

dlab180_Supplementary_Data
