# Supplementary material for: C-reactive protein-guided use of procalcitonin in COVID-19
Source: JAC Antimicrob Resist. 2021 Nov 28;3(4):dlab180. doi: 10.1093/jacamr/dlab180 (PMC8633792; doi:10.1093/jacamr/dlab180)
Supplement: dlab180_Supplementary_Data [file dlab180_supplementary_data.docx]

**Supplementary data**

**Figure S1**


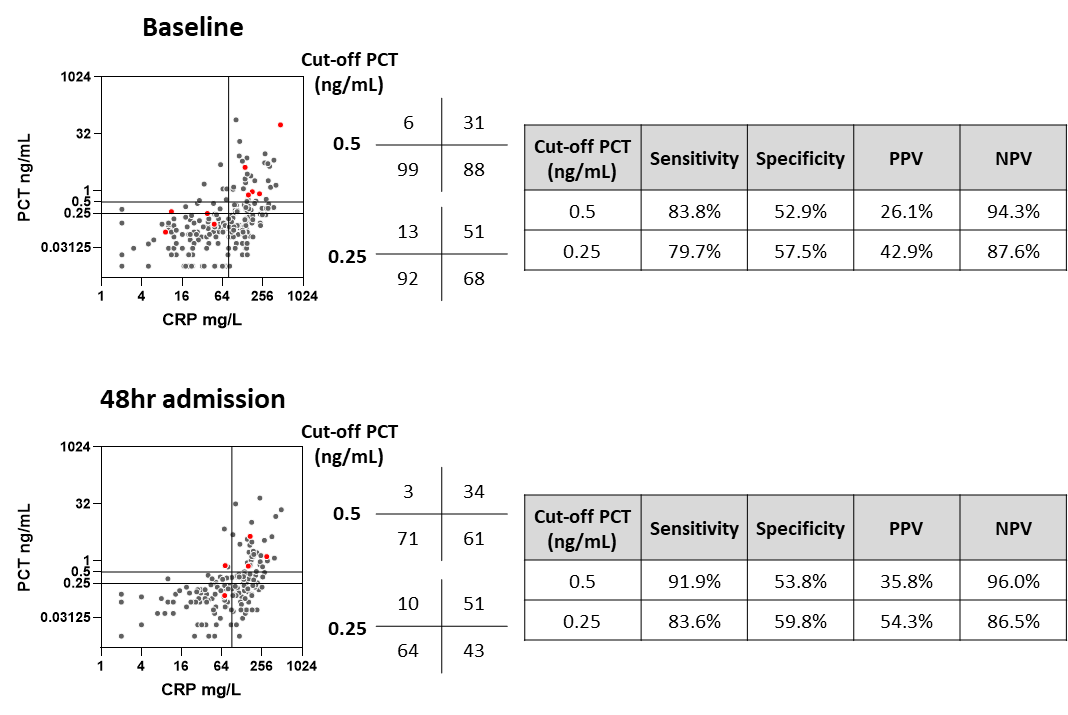


**Figure S1.** Relationship between PCT and CRP concentrations. Scatter plot horizontal lines represent PCT concentration cut-off (≥0.25 or ≥0.5ng/mL) and vertical lines represent median CRP for all patients at each timepoint (79 & 91 mg/L respectively). Total number of patients in each quadrant of scatter plots quantified in the adjacent table. Red dots reflect patients with significant microbiological findings. Sensitivity, specificity, PPV and NPV given derived for elevated PCT determined by CRP concentrations was derived at each timepoint for each PCT cut-off. Assessments were made at the time of hospital admission (“baseline”) or 48 hours into hospital admission (“48hr admission”).
